# Analysis of critical residues for peroxygenation and improved peroxygenase activity via *in situ* H_2_O_2_ generation in CYP105D18

**DOI:** 10.3389/fmicb.2023.1296202

**Published:** 2023-12-11

**Authors:** Bashu Dev Pardhe, Tae-Jin Oh

**Affiliations:** ^1^Department of Life Science and Biochemical Engineering, Graduate School, SunMoon University, Asan, Republic of Korea; ^2^Genome-Based BioIT Convergence Institute, Asan, Republic of Korea; ^3^Department of Pharmaceutical Engineering and Biotechnology, SunMoon University, Asan, Republic of Korea

**Keywords:** CYP105D18, glucose oxidase, mutagenesis, peroxygenase activity, testosterone

## Abstract

Limited numbers of CYPs have been reported to work naturally as peroxygenases. The peroxide shunt pathway can be efficiently used as an alternative for the NAD(P)H and reductase systems, particularly in high hydrogen peroxide (H_2_O_2_) resistance CYPs. We reported the structural and biochemical features of CYP105D18 peroxygenase for its high H_2_O_2_ tolerance capacity. Q348 was a crucial residue for the stability of CYP105D18 during the exposure to H_2_O_2_. In addition, the role of the hydrophilic amino acid T239 from the I helix for peroxygenation and regiospecificity toward testosterone was investigated. Interestingly, T239E differs in product formation from wild type, catalyzing testosterone to androstenedione in the presence of H_2_O_2_. The other variant, T239A, worked with the Pdx/Pdr system and was unable to catalyze testosterone conversion in the presence of H_2_O_2_, suggesting the transformation of peroxygenase into monooxygenase. CYP105D18 supported the alternative method of H_2_O_2_ used for the catalysis of testosterone. The use of the same concentration of urea hydrogen peroxide adducts in place of direct H_2_O_2_ was more efficient for 2β-hydroxytestosterone conversion. Furthermore, *in situ* H_2_O_2_ generation using GOx/glucose system enhanced the catalytic efficiency (*k*_cat_/*K*_m_) for wild type and F184A by 1.3- and 1.9-fold, respectively, compared to direct use of H_2_O_2_ The engineering of CYP105D18, its improved peroxygenase activity, and alteration in the product oxidation facilitate CYP105D18 as a potential candidate for biotechnological applications.

## Introduction

Cytochromes P450 (CYP or P450) are useful candidates for the activation of C-H groups in pharmaceuticals and fine chemicals. Major CYPs require electron transport proteins and expensive reducing equivalents that hamper the use of many CYPs as biocatalysts for industrial applications. The investigation and use of peroxygenase reactions catalyzed by CYPs can be an alternative industrial adaptation for drug modification (Albertolle and Peter Guengerich, 2018; Wei et al., 2018). These peroxygenases use “peroxide shunt” as an alternative to NAD(P)H-driven (reduced nicotinamide adenine dinucleotide phosphate) redox partners to generate catalytically active oxyferryl species using H_2_O_2_ or other organic surrogates (Matthews et al., 2017). In particular, prokaryotic P450s have been mostly investigated to utilize H_2_O_2_ as a co-substrate for the metabolism of specific substrates (Albertolle and Peter Guengerich, 2018).

The CYP152 family of bacterial enzymes is mostly investigated for their ability to modify fatty acids through H_2_O_2_-mediated reactions (Munro et al., 2018); however, CYP116B5, CYP154C8, and CYP105D18 are also reported as peroxygenases (Dangi et al., 2018; Ciaramella et al., 2020; Pardhe et al., 2021). The use of these peroxygenases may be economically viable, but it can also cause heme oxidation and enzyme deactivation, ultimately leading to poor catalytic performance (Munro et al., 2018; Ciaramella et al., 2020). Regardless, the “peroxide shunt” pathway can be efficiently used as an alternative for the NADPH and reductase system, particularly in high H_2_O_2_ resistance CYPs (Dangi et al., 2018; Pardhe et al., 2021). The molecular dynamics (MD) simulation and site-directed mutagenesis approach were used to define the underlying mechanism of redox sensitivity of the human drugs metabolizing CYPs. In CYP4B1, after oxidizing C448 to sulfenic acid (Cys-SOH), the Q451 forms an NH-π bond with F441 to adopt a close conformation, which limits the exposure to oxidizing agents and stabilizes and protects heme–thiolate sulfenic acid. Further mutation to Q451 shows the loss of activity in the presence of the oxidizing agent, H_2_O_2_, and produces a redox-insensitive enzyme (Albertolle et al., 2019).

Limited numbers of CYPs have been reported to study naturally as peroxygenases. The conversion of CYP heme monooxygenase to peroxygenase by introducing site-directed mutagenesis, threonine to glutamate, has been reported (Shoji et al., 2016; Coleman et al., 2020; Podgorski et al., 2022). Direct use of H_2_O_2_ co-substrate for the peroxygenase CYPs results in early heme oxidation and poor catalytic performance. Alternative ways of using H_2_O_2_ in the reaction system have been reported to optimize the heme oxidation rate and increase the catalytic efficiency of a peroxygenase. For example, employing an equimolar concentration of urea hydrogen peroxide (UHP), an adduct that gradually releases H_2_O_2_ in the reaction mixture, results in better total turnover than the direct use of H_2_O_2_ (Lee et al., 2022). Furthermore, the development of CYP peroxygenase by optimizing the H_2_O_2_ production rate in the reaction mixture can minimize the oxidation rate of heme (Oktavia et al., 2023). The use of a supportive enzyme system for H_2_O_2_ generation has shown better catalytic performance in certain CYP peroxygenases (Ciaramella et al., 2019; Giuriato et al., 2022; Oktavia et al., 2023). The P450SPα from *Sphingomonas paucimobilis* was fused with monomeric sarcosine oxidase to create H_2_O_2_-self-producing CYP (Matthews et al., 2017; Giuriato et al., 2022). This fused protein was highly purified and was successfully controlled for the stoichiometric production of H_2_O_2_, leading to a reduced heme decay rate (*k*) and a greater conversion yield toward fatty acids compared to wild type (Giuriato et al., 2022). Recently, self-sufficient CYP102A1 from *Bacillus megaterium* was reported for the peroxygenase reaction using glucose oxidase for the *in situ* generation of H_2_O_2_ for atorvastatin hydroxylation (Oktavia et al., 2023).

In this study, we aimed to investigate the underlying mechanism of high H_2_O_2_ resistance by CYP105D18 from *Streptomyces laurentii* for testosterone bioconversion. The stability of CYP105D18 toward H_2_O_2_ was explored by mutagenesis based on structural features. The polar amino acid T239 present in the active site has been identified as crucial for product distribution and the alternative use of redox partners. The alternative method of H_2_O_2_ use by CYP105D18 for testosterone conversion was applied.

## Materials and methods

### Chemicals and reagents

Glucose oxidase (GOx) from *Aspergillus niger* was purchased from Sigma-Aldrich as a lyophilized solid with an activity of 10 KU. The hydrogen peroxide (H_2_O_2_), urea hydrogen peroxide (UHP), α-aminolevulinic acid (ALA), ampicillin (Amp), nicotinamide adenine dinucleotide (NADH), catalase, formate dehydrogenase, and sodium formate were obtained from Sigma-Aldrich (Korea). Isopropyl-1-thio-β-D-galactopyranoside (IPTG) and kanamycin were bought from Duchefa Biochemie (Korea). Restriction enzymes were purchased from Takara Clontech (Korea).

### Cloning and overexpression of CYPs/mutants and redox partners

CYP105D18 from the strain *Streptomyces laurentii* was cloned, overexpressed, and purified as described in our previous study (Pardhe et al., 2021). Redox partners putidaredoxin reductase (Pdr) (*camA*) and putidaredoxin (Pdx) (*camB*) (P450cam system) from *Pseudomonas putida* were expressed as His-tagged proteins in *E. coli* BL21(DE3) using the plasmid constructs pET28a(+) and pET32a(+) according to the previously described method (Bhattarai et al., 2013). Cells were harvested by centrifugation (9,469 *g*) for 30 min at 4°C, washed twice with a 50 mM potassium phosphate buffer (pH 7.4), and stored at −50°C.

### Site-directed mutagenesis

Mutagenesis was performed using the QuikChange II site-directed mutagenesis kit method as described in our previous study (Pardhe et al., 2023). Q348 in CYP105D18 was mutated to observe its role in thiol sensitivity during exposure to H_2_O_2_. Furthermore, active site residues of CYP105D18 involved in steroid biotransformation were mutated. All primers were designed to anneal at 55°C, and these are listed in Supplementary Table S1. The confirmed mutants were transformed into *E. coli* C41 (DE3) and overexpressed as described in our previous study (Pardhe et al., 2023).

### Determination of enzyme concentrations

The concentration of CYP105D18 and its variants was determined by the carbon monoxide (CO) difference spectra, as described previously (Pardhe et al., 2021). The amount of CYP was calculated from _Ɛ449–489_ = 91 mM^−1^ cm^−1^ (Omura and Sato, 1964). The Pdr concentration was determined as the average concentration calculated from wavelengths 378, 454, and 480 nm using extinction coefficients (_Ɛ_) of 9.7, 10.0, and 8.5 mM^−1^ cm^−1^, respectively, and the Pdx concentration was also determined as the average of concentrations calculated from wavelengths 415 and 454 nm using extinction coefficients of 11.1 and 10.4 mM^−1^ cm^−1^, respectively (Purdy et al., 2004). All samples were scanned using a Biochrom Libra S35PC UV/visible spectrophotometer (Cambridge, United Kingdom).

### Peroxygenase assays

**SCHEME 1:**
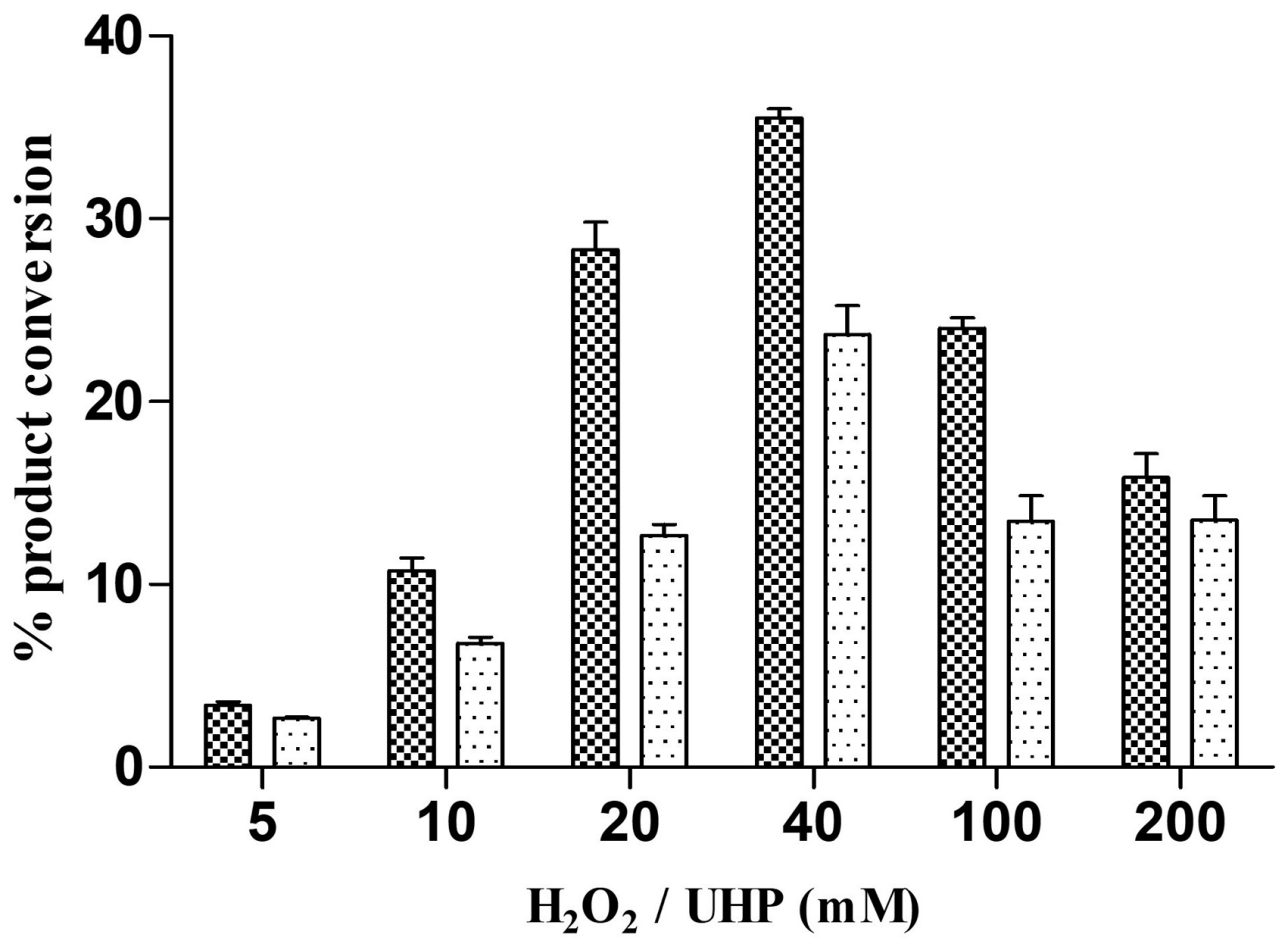
Use of UHP and H_2_O_2_ generation using GOx from *Aspergillus Niger* by CYP105D18 peroxygenase for the catalysis of testosterone.

CYP105D18 peroxygenase activity was compared using direct H_2_O_2_, H_2_O_2_ adduct (UHP), and H_2_O_2_ generation (GOx/glucose) (Scheme 1). The peroxygenase oxidation assays were run at 30°C in a total volume of 400 μL potassium phosphate buffer (pH 7.4, 50 mM). The reaction mixture consists of 2 μM enzyme and 200 μM substrate with different concentrations of H_2_O_2_ and UHP. The reaction was started by adding H_2_O_2_ (5/10/20/40/100/200 mM) or UHP (5/10/20/40/100/200 mM) and incubated for 1 h at 700 rpm. UHP was added as a solid powder, while H_2_O_2_ was added from a 1-M stock solution. A stock solution of 1,000 U/mL for GOx was prepared in potassium phosphate buffer, which was determined by the glucose oxidase activity assay kit. A total volume of 400 μL potassium phosphate buffer consisting of 3 μM enzyme, 200 μM substrate (4/8/16/32/48 U) GOx, and a reaction mixture was initiated by 6.6 mM glucose and incubated for 1 h at 700 rpm at 30°C. The glucose concentration for H_2_O_2_ generation was optimized using a range of 2–80 mM at a fixed GOx. The reaction mixture contained CYP, Pdr, and Pdx (1:2:16 ratio), catalase (100 mg/mL), MgCl_2_ (1 mM), substrate (200 μM), and the NADH regeneration system (1 U formate dehydrogenase and 150 mM sodium formate). The reaction was initiated by NADH and incubated for 2 h at 30°C with vigorous shaking at 700 rpm.

### Heme bleaching assay

A 1-mL aliquot of CYP105D18, Q348L mutant, or Q348E mutant (3 μM) was prepared in a 50-mM potassium phosphate buffer pH 7.4. The heme oxidation rate was determined using H_2_O_2_ concentrations of 20/40/80 mM. After adding H_2_O_2_, the absorbance was recorded at a wavelength of 350–500 nm every 90 s for 30 min. The Soret peak absorbance at 417 nm was plotted against time, and the destruction of heme was measured by calculating the heme oxidation rate constant (*k*) using one-phase decay in GraphPad Prism 5 software. Associated absorbance amplitudes (A) were calculated as the differences between the highest and lowest absorbance at 417 nm. All samples were scanned using a Biochrom Libra S35PC UV/visible spectrophotometer.

### Product analysis

The reactions were extracted using an equal volume of ethyl acetate twice, dried, and dissolved in HPLC-grade methanol for further analysis. The mixture was filtered using a 0.2-μm Whatman filter, injected into an ultra-HPLC instrument, and separated with the use of the Mightysil reverse-phase C18 column (4.6 × 250, 5 μm; Kanto Chemical, Tokyo, Japan). The gradient of acetonitrile (B) was maintained at 10% for 10 min and gradually increased to 50% from 10 to 20 min, 70% from 20 to 25 min, and then gradually decreased to 15% from 25 to 40 min. Water was used as solvent A. Testosterone and its product was detected by UV-A at 242 nm. Product androstenedione and 2β-hydroxytestosterone were correlated with standard retention time in the HPLC chromatogram (Pardhe et al., 2023). Following the HPLC analysis, the reaction mixtures were analyzed using SYNAPT G2-S/ACUITY UPLC liquid-chromatography quadrupole time-of-flight/electrospray ionization mass spectrometry (Waters, Milford, MA, United States) in positive ion mode.

### Docking analysis

CYP105D18 (Pdb 7di3) was obtained from the Protein Data Bank.^1^ Structural optimization of testosterone and rigid formation molecular docking was performed using Gnina. The grid box was set up (ADT ver.1.5.6)^2^ to include HEM, and the box size was set to 35 Å × 35 Å × 30 Å, spacing of 0.375 Å, and locations of x = 14.721, y = −3.573, and z = 14.768. The whole docking simulation parameters were set to num_modes 500, exhaustiveness = 8, and min_rmsd_filter = 0.5. The protein–ligand visualization and structural figures were prepared using PyMOL (DeLano, 2002).

### Kinetics analysis

Testosterone oxidation rates by CYP105D18 and its mutant were calculated from the initial 5-min reaction using 1 μM CYP, 40 mM H_2_O_2_ or 8 U GOx, and 36 mM glucose in phosphate buffer pH 7.4 at 30°C. The substrate inhibition kinetics was applied to determine the kinetic parameters for testosterone biotransformation by CYP105D18 and its mutant. Assuming the non-competitive enzyme inhibition occurs when the substrate concentration is high, the Michaelis–Menten substrate inhibition equation {v = *V*_max_[S]/(*K*_m_ + [S]х(1 + [S]/*K*_i_))} (empirical model) was applied to characterize the substrate inhibition kinetics (Wu, 2011; Kratky et al., 2023). *V*_max_ and *K*_m_ are defined as the maximum velocity and substrate concentration at which the velocity is equal to half of the maximum velocity, respectively. *K*_i_ is the substrate inhibition constant (Wu, 2011). Furthermore, *K*_m_, *K*_i,_ and *k*_cat_ values were calculated by plotting the product formation rate against substrate concentration.

## Results

### Structural features of CYP105D18 enabling the stability for H_2_O_2_

**Figure 1:**
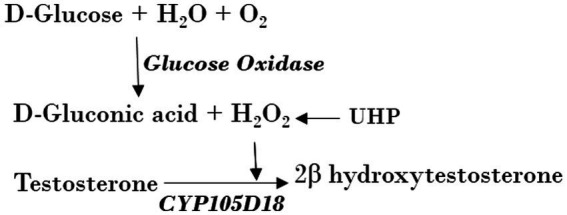
**(A)** Superposition of CYP105D18 with H_2_O_2_ insensitive rabbit P450 4B1. Residues from CYP105D18 corresponding to C448, F441, and Q451 of P450 4B1 are shown using sticks. **(B)** Molecular docking of testosterone with CYP105D18. Testosterone is shown in yellow color. The distance between C-2 and heme was calculated.

**Figure 2:**
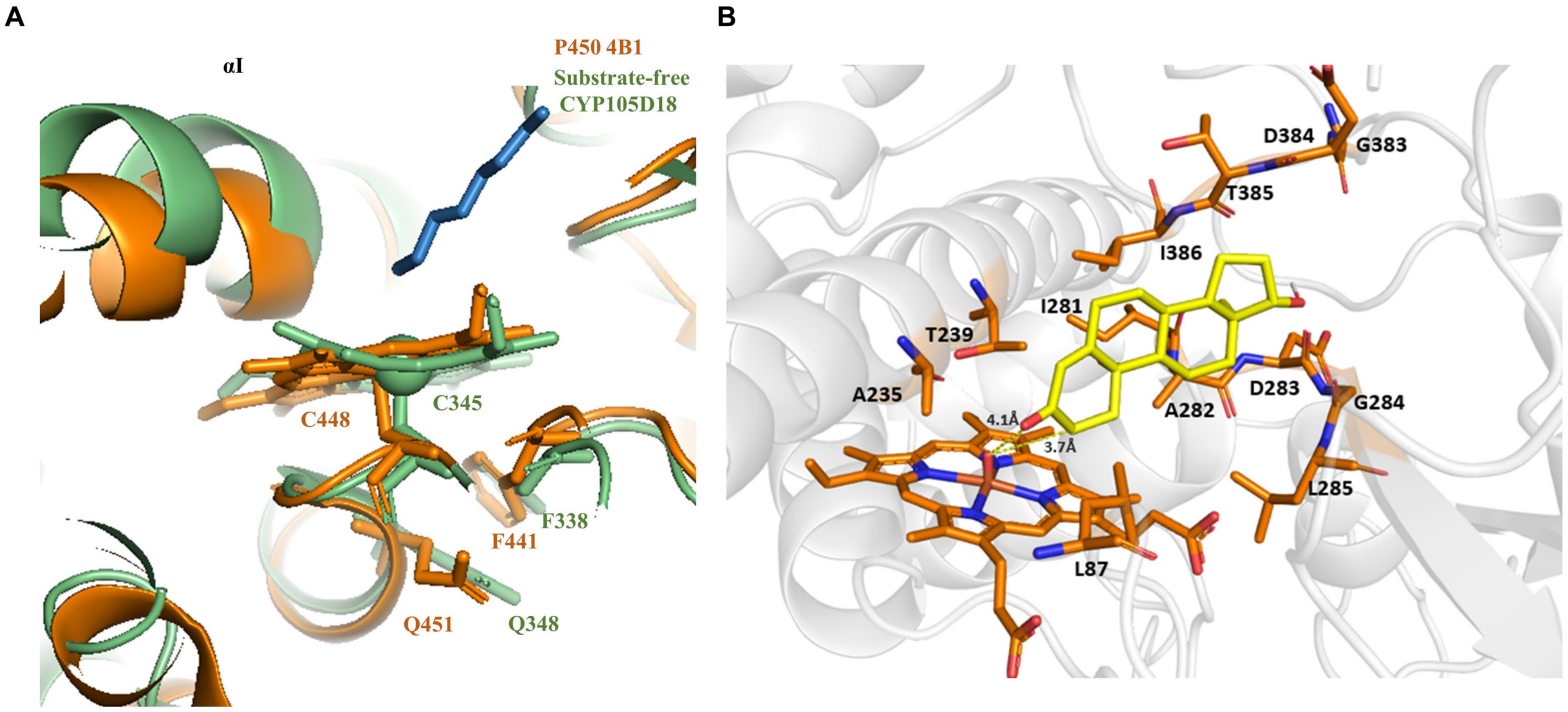
Heme oxidation pattern for CYP105D18 **(A)**, and its mutant Q348L **(B)**, and Q348E **(C)**, Heme oxidation rate was calculated for 30 min at 90 S intervals in the presence of 40 mM H_2_O_2_. “*k*” is the heme dissociation constant presented with mean ± standard error. **(D)** The respective bar diagrams represent the formation of 2β-hydroxytestosterone in the presence of 40 mM H_2_O_2_, 200 μM testosterone, and 1 μM CYP. Statistical analysis was performed using an ordinary one-way ANOVA test, followed by Dunnett’s multiple comparison test with a statistically significant value set at *p* < 0.05 (*).

The investigation of the crystal structure of CYP105D18 showed a similar structural feature for H_2_O_2_ resistance to rabbit P450 4B1 (Albertolle et al., 2019). The superposition of CYP105D18 with P450 4B1 displays the conserved residue that is involved in the P450 4B1 to adopt conformational change that may stabilize and protect heme–thiolate sulfenic acid. C448, F441, and Q451 from P4504B1 are conserved in CYP105D18, corresponding to C345, F338, and Q348, respectively (Figure 1A). In CYP4B1, after oxidizing C448 to sulfenic acid (Cys-SOH), the Q451 forms an NH-π bond with F441 to adopt a close conformation, which protects heme from the oxidants. We hypothesized a similar mechanism for CYP105D18 and prepared the mutant of Q348. Mutation of isosteric leucine (Q348L) and glutamate (Q348E) would increase the H_2_O_2_ sensitivity. Furthermore, heme oxidation rate and % conversion for β-hydroxy testosterone using 40 mM H_2_O_2_ were measured for wild type and mutants. Heme oxidation rates were higher in both Q348L (*k* = 0.0213 ± 0.0008 min^−1^) and Q348E (*k* = 0.0277 ± 0.0003 min^−1^) mutants compared to wild type (*k* = 0.0137 ± 0.0003 min^−1^), indicating a decrease in H_2_O_2_ tolerance capacity. Similarly, % conversion was significantly lower in both mutants compared to the wild type, which enables the characteristic feature of Q348 for the stability of CYP105D18 for H_2_O_2_ (Figure 2).

### Site-directed mutagenesis of the active site for testosterone biotransformation

**SCHEME 2:**
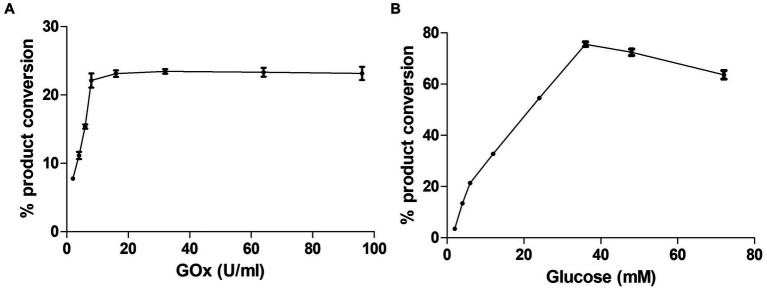
Reactions catalyzed by CYP105D18 and its variants with a combination of different redox partners. Highlighted system in the reaction arrow represents the catalyzed reaction whereas the dimmed system represents no catalyzed reaction.

The molecular docking analysis of testosterone with CYP105D18 demonstrated that testosterone was surrounded by the hydrophilic residue T239 along with all the other hydrophobic residues L87, A235, I281, A282, D283, L285, and I386 (Figure 1B). The exposure of the A-ring facing the 2C toward the heme and the role of this hydrophilic amino acid T239 in the binding and accommodation of testosterone may be crucial for regiospecific biotransformation (Zhang et al., 2020). A change in the acid–alcohol pair in the I helix has been reported for increased peroxygenase activity (Coleman et al., 2020; Podgorski et al., 2022). In this study, we performed the site-directed mutagenesis of active site residues T239 and A235, which belong to the I helix. Additionally, the B/C loop from CYP105D18 in the active site was flexible and remained in open confirmation without substrate (Pardhe et al., 2021). The active site residue L87 present in the B/C was mutated to understand the role of that amino acid. Mutants L87A, L87E, and A235E did not show any significant changes in the conversion rate of testosterone compared to the wild type. Furthermore, polar residue T239 was mutated into T239A and T239E. Interestingly, variants from the T239 displayed differences in product distribution patterns and utilization in redox partners (Scheme 2). As we reported, CYP105D18 was peroxygenase and was unable to utilize the Pdx/Pdr system for the catalysis (Pardhe et al., 2021, 2023). T239A was able to utilize NADH with the Pdx/Pdr system for the biotransformation of testosterone to 2β-hydroxytestosterone. The T239A variant was unable to catalyze testosterone in the presence of H_2_O_2_, suggesting the transformation of peroxygenase to monooxygenase.

**Figure 3:**
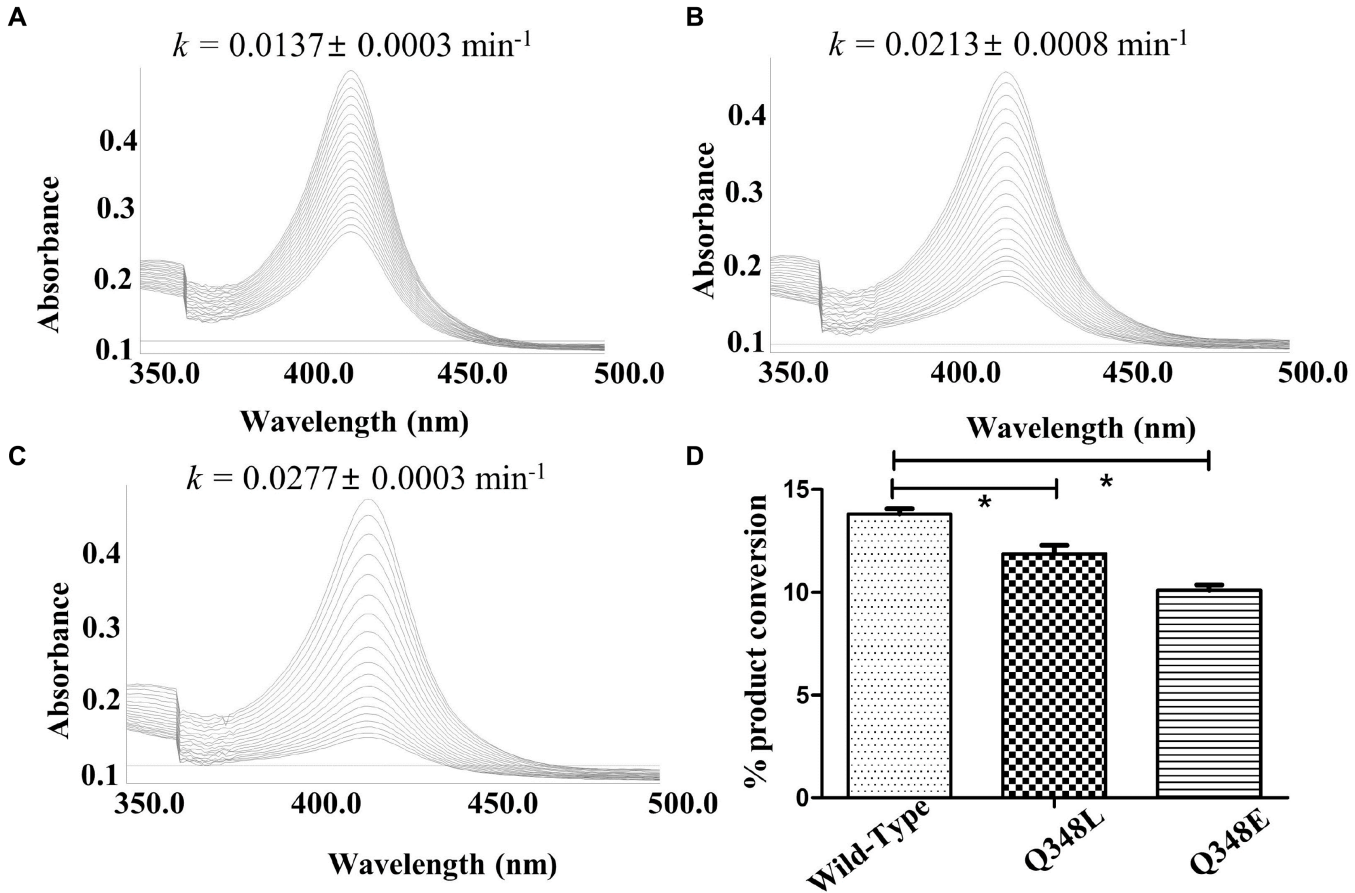
**(A)** HPLC chromatogram for the testosterone biotransformation catalyzed by CYP105D18 and its variants. (i) Testosterone catalyzed by CYP105D18 in the presence of H_2_O_2_. (ii) Standard chromatogram for androstenedione. (iii) Testosterone catalyzed by T239E mutant of CYP105D18 in the presence of H_2_O_2_. P2 is the second product catalyzed by T239E. **(B)** Kinetics analysis for testosterone catalyzed by T239E mutant of CYP105D18. All the reactions were performed using 1 μM CYP and 40 mM H_2_O_2_. *K*_m_, *K*_i_, and *k*_cat_ values were calculated from three independent reactions under similar conditions.

The other variant, T239E, catalyzed testosterone to androstenedione in the presence of H_2_O_2_, which was a different product from the wild type (Scheme 2; Supplementary Figure S1). Previously, we reported that androstenedione was the substrate for CYP105D18 wild type with single and double-hydroxylated products. T239E catalyzed testosterone to androstenedione (retention time (Rt) 18.3 min), and negligible double-hydroxylated androstenedione (Rt 13.2 min) was also detected (Figure 3A). Furthermore, T239 was changed to other amino acids with a hydrophobic side chain. T239M, T239I, and T239G catalyze testosterone to 2β-hydroxytestosterone using H_2_O_2,_ and the conversion rates were lower than wild-type enzymes (Supplementary Figure S2). T239V was unstable during the purification and showed no activity toward testosterone using any redox system or H_2_O_2_.

### Alternative ways of H_2_O_2_ generation to yield the catalytic efficiency of the peroxygenase CYP105D18

**Figure 4:**
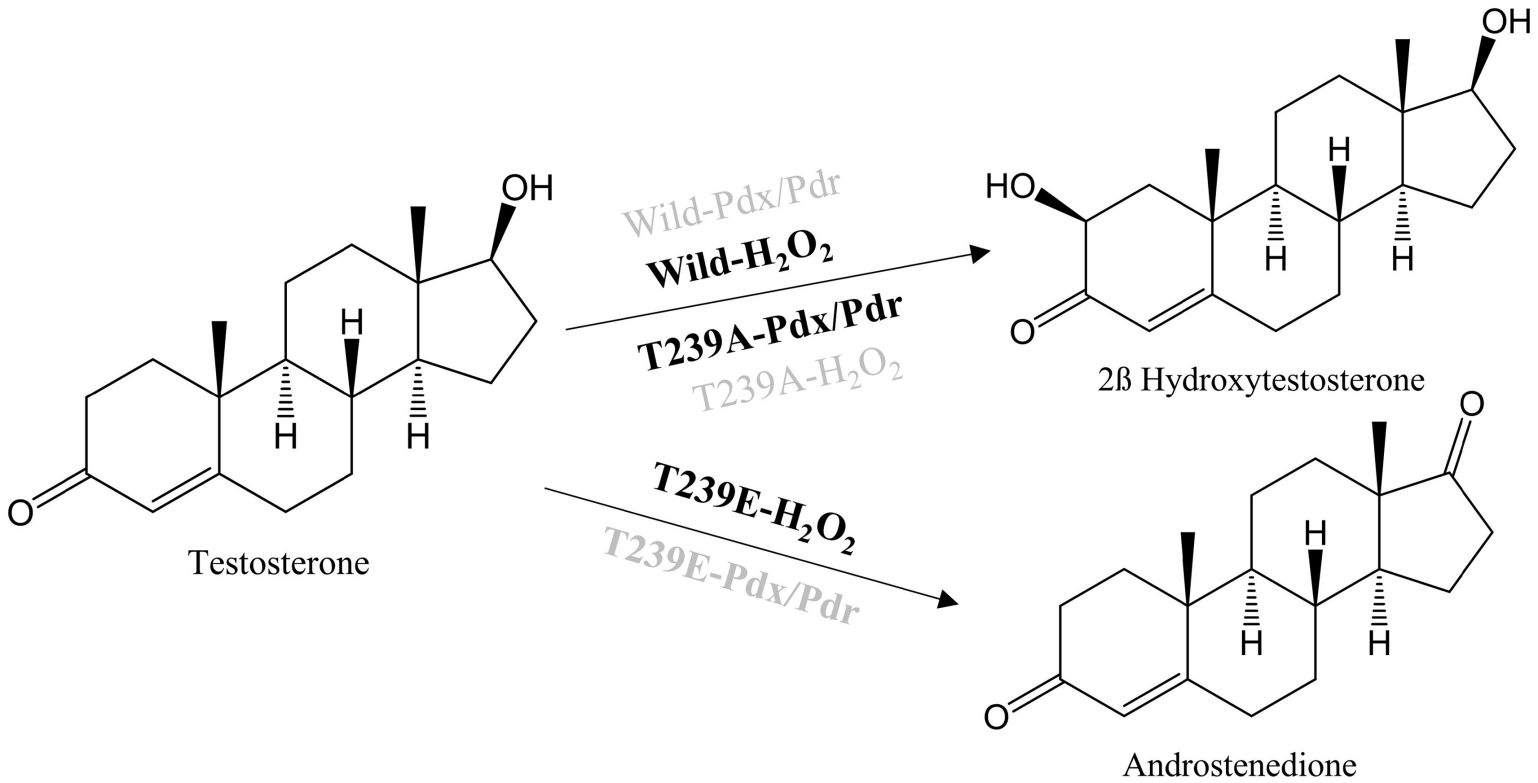
Comparison of % conversion of 2β-hydroxytestosterone by CYP105D18 using an equimolar concentration of UHP (dark) and H_2_O_2_ (light). All experiments were conducted using 2 μM CYP105D18 and 200 μM testosterone at 30°C for 1 h. The reactions were conducted in triplicate under similar conditions.

**Figure 5:**
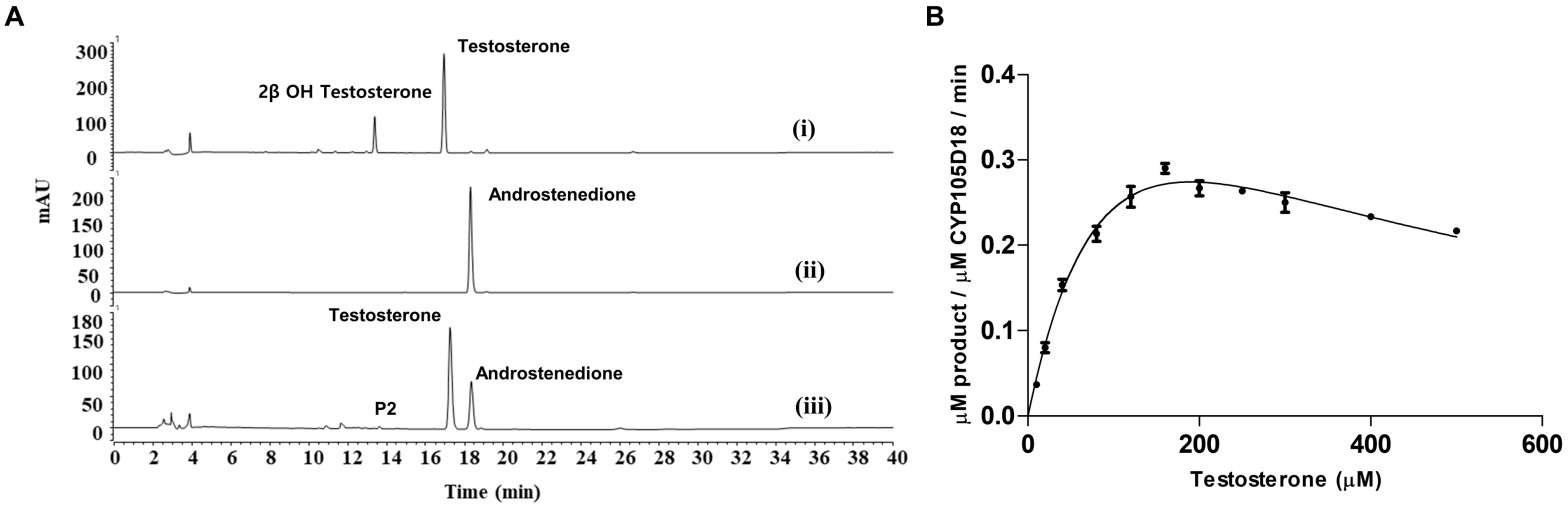
Optimization of testosterone catalysis by CYP105D18 using GOx/glucose system. **(A)** Optimization of GOx concentration for testosterone catalysis at a fixed amount of glucose to be 6.6 mM. **(B)** Optimization of glucose concentration for testosterone catalysis at a fixed amount of GOx to be 8 U/mL. All experiments were conducted using 3 μM CYP105D18 and 200 μM testosterone at 30°C for 1 h. The reactions were conducted in triplicate under similar conditions.

CYP105D18 utilizes H_2_O_2_ well for the hydroxylation of steroids. Even though it possesses a high H_2_O_2_ tolerance capacity, direct use of H_2_O_2_ degrades the heme faster and diminishes the catalytic performance. In this study, we use the same concentration of urea hydrogen peroxide adduct in place of using H_2_O_2_ directly. The optimum H_2_O_2_ concentration for testosterone hydroxylation was 40 mM with ≈25% conversion by 2 μM CYP. Using 40 mM UHP, we achieved ≈36% conversion of 2β-hydroxytestosterone (Figure 4). In this study, we tested H_2_O_2_ generation using GOx/glucose to find a better condition to support CYP105D18 for testosterone biotransformation. Initially, we limited the glucose concentration to 6.6 mM and observed the GOx range from 2 to 100 U/mL for the highest turnover for testosterone. We achieve the highest % conversion of testosterone to 2β-hydroxytestosterone at 8 U/mL. Then, we tested the optimal conditions for the reaction using glucose concentrations in the range of 2–80 mM, using a fixed GOx of 8 U/mL. We achieved ≈75% conversion of 2β-hydroxytestosterone using 36 mM glucose, 8 U/mL GOx, and 3 μM CYP105D18 (Figure 5).

### Kinetics for testosterone by CYP105D18/mutants using GOx/glucose H_2_O_2_ generation system

In our previous results, the catalytic efficiency for 2β-hydroxytestosterone formation using direct H_2_O_2_ by F184A was higher than the wild type. Kinetic parameters for 2β-hydroxytestosterone formation were calculated using the optimized conditions for H_2_O_2_ generation for wild type and F184A mutants. A high concentration of substrate was found to inhibit enzyme activity, and the data obtained from the experiments best fit the substrate inhibition model. The inhibitory potency of a substrate (*K*_i_) was calculated assuming that substrate inhibition occurs when two molecules of a substrate bind to the enzyme at its increased concentration. *K*_m_, *K*_i,_ and *k*_cat_ values for the wild type were 129.9 ± 17.8 μM, 494 ± 79.2 μM, and 0.79 ± 0.06 min^−1^, respectively. Whereas, *K*_m_, *K*_i_, and *k*_cat_ values for F184A were 140.4 ± 15.3 μM, 364 ± 44.2 μM, and 1.7 ± 0.1 min^−1^, respectively (Supplementary Figure S3). The catalytic efficiency (*k*_cat_/*K*_m_) was increased for wild type and F184A by 1.3- and 1.9-fold, respectively, using H_2_O_2_ generation compared to the direct use of H_2_O_2_ (Table 1).

**Table 1:** Comparison of kinetic parameters between direct use of H_2_O_2_ and H_2_O_2_ generation systems by CYP105D18 and its mutant for testosterone biotransformation.

	*K*_m_ (μM)	*k*_cat_ (min^−1^)	*K*_i_ (μM)	*k*_cat_/*K*_m_ (μM^−1^S^−1^)	*K*_i_/*K*_m_	References
T239E/H_2_O_2_	133.8 ± 28.6	0.60 ± 0.09	278.4 ± 67.8	0.27	2.0	This study
Wild type/H_2_O_2_	127.0 ± 40.0	0.60 ± 0.10	465.0 ± 188.0	0.28	3.6	Pardhe et al. (2023)
F184A/H_2_O_2_	158.0 ± 37.0	1.00 ± 0.16	240.0 ± 61.0	0.38	1.5	Pardhe et al. (2023)
Wild type/GOx	129.9 ± 17.8	0.79 ± 0.06	494.0 ± 79.2	0.36	3.8	This study
F184A/GOx	140.4 ± 15.3	1.70 ± 0.10	364.0 ± 44.2	0.73	2.6	This study

GOx, glucose oxidase; H_2_O_2_, hydrogen peroxide.

T239E was able to catalyze testosterone to androstenedione in the presence of H_2_O_2_. Therefore, we performed the kinetic parameters for androstenedione formation, and the experimental data generated best fit with the substrate inhibition kinetics similar to those of the wild-type enzyme (Pardhe et al., 2023; Figure 3B). *K*_m_, *K*_i_, and *k*_cat_ values were 133.8 ± 28.6 μM, 278.4 ± 67.8 μM, and 0.6 ± 0.09 min^−1^, respectively. T239E showed higher testosterone inhibition compared to the wild-type enzyme, which has a *K*_i_ value of 465 ± 188 μM for 2β-hydroxytestosterone formation.

## Discussion

CYP105D18 peroxygenase catalyzes H_2_O_2_-dependent hydroxylation of steroids; therefore, we investigated the H_2_O_2_ tolerance capacity by calculating the heme degradation rate (*k*). Unexpectedly, CYP105D18 demonstrated high tolerance for H_2_O_2_, as the “*k*” was low even in the presence of higher H_2_O_2_. The well-established P450 peroxygenase P450s OleT (CYP152L1 from *Jeotgalicoccus* sp.), BSβ (CYP152A1 from *Bacillus subtilis*), and SPα (CYP152B1 from *Sphingomonas paucimobilis*) have been found to substitute the acid–alcohol amino acid pair to enable proton relay to iron-oxo species and assist in the catalytic cycle (Munro et al., 2018). In our previous study, we reported that the steroid hydroxylase CYP154C8 monooxygenase functioned unexpectedly in the presence of a high H_2_O_2_ concentration (Dangi et al., 2018). The catalytic efficiency of CYP relies significantly on its heme stability. CYP105D18 peroxygenase showed higher stability of heme, showing a lower *k*-value than those reported peroxygenases, CYP154C8, CYP152L1, and P450116B5hd (Pardhe et al., 2021).

Furthermore, the possible mechanism of H_2_O_2_ resistance for CYP105D18 was investigated using structural features and site-directed mutagenesis. H_2_O_2_-mediated P450 inactivation occurs either through heme release from the heme-binding site or the destruction of heme. The H_2_O_2_-mediated self-inactivation of cytochrome P450 2B4 during benzphetamine oxidation was accompanied by heme degradation and apoenzyme modification (Karuzina et al., 1999). H_2_O_2_ can also modify some amino acid residues, including the oxidation of heme–thiolate ligand to sulfenic acid, which can further cause the release of heme from CYP, thus inhibiting the catalysis, which was first identified with P450 4A11 (Karuzina et al., 1999; Albertolle et al., 2017; Albertolle and Peter Guengerich, 2018). In another case, human P450 1A2 was reported as thiol-insensitive to H_2_O_2_, where hyperoxidation of ancillary cysteine C159 did not affect the catalysis. This phenomenon may be related to the stabilization of the heme–thiolate from oxidants due to the overall structural features of the protein or the heme surrounding amino acids (Albertolle et al., 2018). Q451 of rabbit P450 4B1 was reported as an essential residue for thiol sensitivity (Albertolle et al., 2019). Q348, which was also conserved in CYP105D18 such as Q451 in P450 4B1, was mutated, and thiol sensitivity was analyzed in the presence of H_2_O_2_. Both the mutant Q348L and Q348E increase the redox sensitivity in CYP105D18. In CYP105D18, after oxidizing C345 to sulfenic acid (Cys-SOH) by H_2_O_2_, the Q348 may form an NH-π bond with F338 to adopt a close conformation, which further protects thiol oxidation and subsequently heme oxidation. This feature may enable CYP105D18 to exhibit more catalytic efficiency before definitive heme oxidation.

Conserved acid–base pairs (histidine, aspartate, or glutamate) present in the heme-containing peroxygenase and absent from P450s are responsible for the generation of compound I, which is responsible for the monooxygenation of a substrate (Shoji and Watanabe, 2014). The I helix, responsible for the heme-binding domain, is conserved in CYP105D18 peroxygenase. T239 in the heme-binding domain was responsible for the accommodation of testosterone and may be crucial for its regiospecific biotransformation. A change in this residue to an acid–base residue is expected to enhance the peroxygenase activity, which was displayed by P450cam, P450BM3, and CYP119 (Shoji et al., 2016). Recently, a mutation on the corresponding amino acid T252 to acidic residue (T252E) in CYP199A4 was able to switch this monooxygenase to peroxygenase (Podgorski et al., 2022). The peroxygenase activity of the T239E variant from CYP105D18 showed an interesting result by catalyzing testosterone to a different product androstenedione. This indicates that T239 is also responsible for the regiospecific transformation of testosterone. The catalytic efficiency of CYP105D18 for 2β-hydroxytestosterone formation and the catalytic efficiency of T239E for androstenedione formation were almost similar, showing that T239E was not responsible for enhancing peroxygenase activity in CYP105D18. In another aspect, this conserved threonine was replaced by alanine or valine in P450cam, which resulted in oxidase activity generating H_2_O_2_ with a reduction in substrate oxidation (Imai et al., 1989). Similarly, the CYP199A4 variant T252A was reported to decrease the coupling efficiency of the enzyme (Coleman et al., 2020). T239A from CYP105D18 completely lost the peroxygenase activity, but it was able to use NADH with the Pdx/Pdr system for the 2β-hydroxylation of testosterone. This switch from peroxygenase CYP to monooxygenase is the first report, and additional mechanism investigation is required. Furthermore, we mutated T239 to other similar amino acids with hydrophobic side chains: valine, isoleucine, glycine, and methionine. T239V was unstable during the purification and showed no activity toward testosterone, and T239M, T239I, and T239G catalyzed testosterone to 2β-hydroxytestosterone with a decreased conversion rate using H_2_O_2_, and they were unable to utilize reducing equivalents.

Even though CYP105D18 possesses high H_2_O_2_ tolerance, providing an adequate supply of H_2_O_2_ to minimize oxidative inhibition while providing enough supply to achieve maximum catalytic activity remains challenging. In this study, we applied the use of UHP, which is an adduct of H_2_O_2_ that slowly releases free H_2_O_2_ in an aqueous solution (Ankudey et al., 2006; Ingenbosch et al., 2021). The optimum concentration of UHP was similar to the H_2_O_2_ for CYP105D18, and there was approximately 1.4х increase in % 2β-hydroxytestosterone conversion. Lee, Joel HZ, et al., also reported only moderate differences in the level of product formation using the equimolar concentration of UHP and H_2_O_2_ (Lee et al., 2022). There was no detectable conversion of 6β-hydroxytestosterone from testosterone by CYP3A4 using direct aqueous H_2_O_2_. Alternative uses of the same concentration of UHP and other H_2_O_2_ donors showed product formation. This advantage of slow release of H_2_O_2_ from H_2_O_2_ adducts in the reaction mixture can enhance the catalytic activity compared to the direct use of H_2_O_2_, which could cause early heme oxidation (Chefson et al., 2006).

Furthermore, the direct addition of exogenous H_2_O_2_ can be replaced by using H_2_O_2_ generation systems. Enzymatic systems such as alcohol oxidase (Ni et al., 2016), sarcosine oxidase (Giuriato et al., 2022), alditol oxidase (AldO) (Matthews et al., 2017), and glucose oxidase (Oktavia et al., 2023) have been successfully reported for H_2_O_2_ generation in the chemical reactions catalyzed by CYPs peroxygenase. The optimized condition for the use of the GOx/glucose ratio yields improved catalytic activity by CYP105D18. The direct use of high concentrations of H_2_O_2_ causes heme degradation and diminishes the catalytic efficiency of the enzyme (Chefson et al., 2006). The slow and continuous generation of a stoichiometric amount of H_2_O_2_ by using the GOx/glucose system was well supported by CYP105D18, and catalytic efficiency was increased by 1.3-fold. This indicates that the stability of the heme in P450 remains crucial when using oxidants as its co-substrate. The combination of highly active mutant H120 of CYP102A1 and *in situ* H_2_O_2_ generation system GOx was able to yield the highest total turnover numbers (TTNs) (56.7 ± 0.7) for 4-OH atorvastatin (Oktavia et al., 2023). The multi-enzyme system using lyophilized CRL (lipase)/OleT_JE_/AldO (alditol oxidase) was successfully optimized for H_2_O_2_ generation and production of α-olefin from coconut oil (Jiang et al., 2020). In another approach, the use of the fusion protein P450_SPα_-polyG-MSOX (monomeric sarcosine oxidase) (H_2_O_2_ generation system MSOX was fused with P450SPα) was able to yield much higher TTNs toward fatty acids compared to the isolated P450SPα (Giuriato et al., 2022). The other example is the fusion of AldO with OleT_JE,_ which was able to control H_2_O_2_ supply to minimize heme oxidation, resulting in increased alkane production (Matthews et al., 2017). These simple H_2_O_2_ generation enzymes such as alditol oxidase, glucose oxidase, and sarcosine oxidase benefit from their abundant use of substrate, but their equivalent release of H_2_O_2_ requires a high substrate concentration, which can sometimes be toxic for the reaction systems. Ni et al. proposed the use of an enzymatic cascade where methanol (substrate) can be fully converted to CO_2_, releasing three equivalents of H_2_O_2_, for the potential use of peroxygenase for oxyfunctionalization chemistry (Ni et al., 2016).

Overall, the critical residues responsible for H_2_O_2_ tolerance and regiospecific biotransformation of testosterone in CYP105D18 were investigated based on detailed structural features and mutant analysis. The alternative use of UHP and *in situ* generation of H_2_O_2_ by the GOx/glucose system was successfully applied in CYP105D18 for testosterone hydroxylation. The catalytic efficiency for 2β-hydroxytestosterone was increased by *in situ* H_2_O_2_ generation using the GOx/glucose system compared to direct use of H_2_O_2_. These applied engineering in CYP105D18 enable the exposure and engineering of CYPs from the CYP105 family for more biotechnological applications.

## Data availability statement

The original contributions presented in the study are included in the article/Supplementary material, further inquiries can be directed to the corresponding author.

## Author contributions

T-JO: Conceptualization, Funding acquisition, Investigation, Project administration, Writing – original draft, Writing – review & editing. BP: Data curation, Formal analysis, Investigation, Methodology, Project administration, Writing – original draft, Writing – review & editing.

## Footnotes

^1^ https://www.rcsb.org

^2^ http://mgltools.scripps.edu/
