# Supplementary material for: Analysis of critical residues for peroxygenation and improved peroxygenase activity via in situ H2O2 generation in CYP105D18
Source: Front Microbiol. 2023 Dec 11;14:1296202. doi: 10.3389/fmicb.2023.1296202 (PMC10750395; doi:10.3389/fmicb.2023.1296202)
Supplement: Supplementary file 1 [file Data_Sheet_1.docx]

**Analysis of critical residues for peroxygenation and improved peroxygenase activity via in situ H_2_O_2_ generation in CYP105D18**

Bashu Dev Pardhe^1^ and Tae-Jin Oh^1,2,3*^

^1^ Department of Life Science and Biochemical Engineering, Graduate School, SunMoon University, Asan 31460, South Korea

^2^ Genome-based BioIT Convergence Institute, Asan 31460, South Korea

^3^ Department of Pharmaceutical Engineering and Biotechnology, SunMoon University, Asan 31460, South Korea

*Correspondence: tjoh3782@sunmoon.ac.kr (T.-J. Oh)

**Table S1**. Mutagenesis primers used for active site engineering and H_2_O_2_ tolerance in CYP105D18.

| Primers | Sequences (5’ – 3’) |
| --- | --- |
| L87A_FP | GCCGCGGGGCACTCGCCGGCTGGGACGA |
| L87A_RP | TCGTCCCAGCCGGCGAGTGCCCCGCGGC |
| L87E_FP | GCCGCGGGGCACTCGAAGGCTGGGACGACC |
| L87E_RP | GGTCGTCCCAGCCTTCGAGTGCCCCGCGGC |
| A235E_FP | ACGGTCCTGCTGGTGGAAGGGCACGAGACCACC |
| A235E_RP | GGTGGTCTCGTGCCCTTCCACCAGCAGGACCGT |
| T239A_FP | TGGCGGGGCACGAGGCCACCGCGAACATGAT |
| T239A_RP | ATCATGTTCGCGGTGGCCTCGTGCCCCGCCA |
| T239E_FP | GTGGCGGGGCACGAGGAAACCGCGAACATGAT |
| T239E_RP | ATCATGTTCGCGGTTTCCTCGTGCCCCGCCAC |
| T239I_FP | TGGTGGCGGGGCACGAGATTACCGCGAACATGAT |
| T239I_RP | ATCATGTTCGCGGTAATCTCGTGCCCCGCCACCA |
| T239G_FP | TGGCGGGGCACGAGGGCACCGCGAACATGAT |
| T239G_RP | ATCATGTTCGCGGTGCCCTCGTGCCCCGCCA |
| T239M_FP | TGGTGGCGGGGCACGAGATGACCGCGAACATGAT |
| T239M_RP | ATCATGTTCGCGGTCATCTCGTGCCCCGCCACCA |
| T239V_FP | TGGCGGGGCACGAGGTGACCGCGAACATGAT |
| T239V_RP | ATCATGTTCGCGGTCACCTCGTGCCCCGCCA |
| Q348L_FP | CAGTGCCTGGGGCTGAACCTGGCGCGGATC |
| Q348L_RP | GATCCGCGCCAGGTTCAGCCCCAGGCACTG |
| Q348E_FP | CAGTGCCTGGGGGAAAACCTGGCGCGGATC |
| Q348E_RP | GATCCGCGCCAGGTTTTCCCCCAGGCACTG |
| ^FP. forward primer, RP. reverse primer^ | |


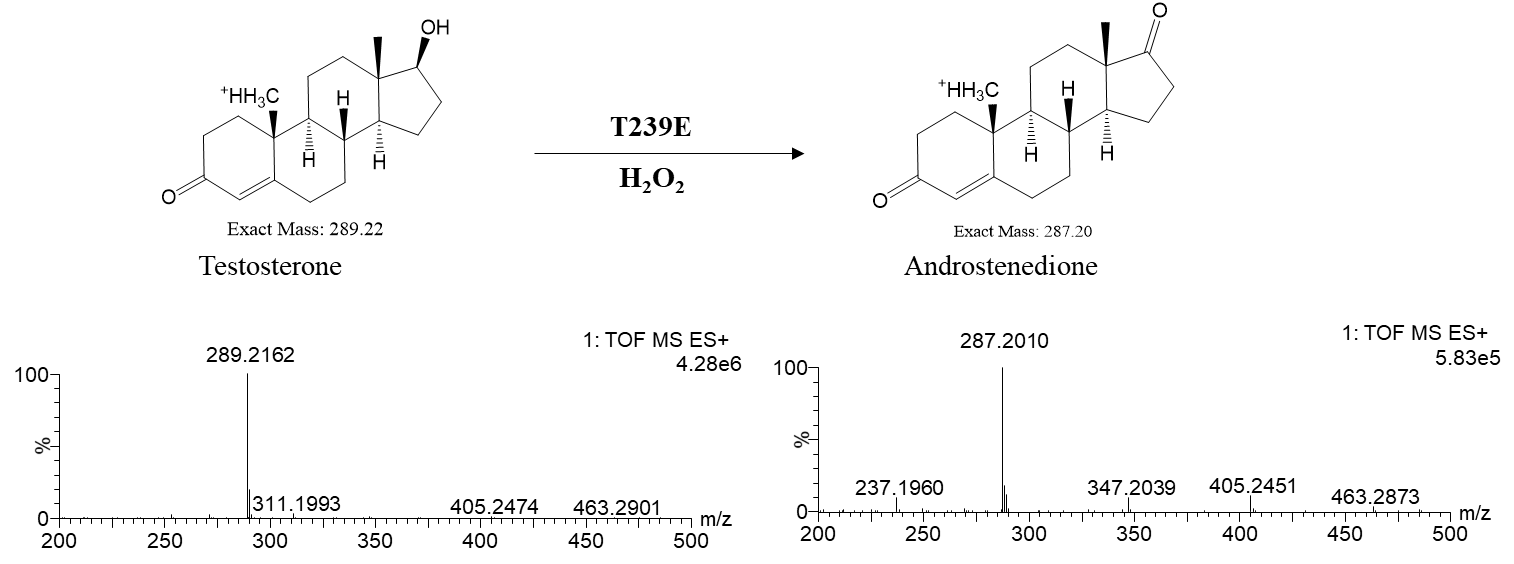


**Figure S1**. Mass data for androstenedione catalyzed by T239E in the presence of H_2_O_2_.


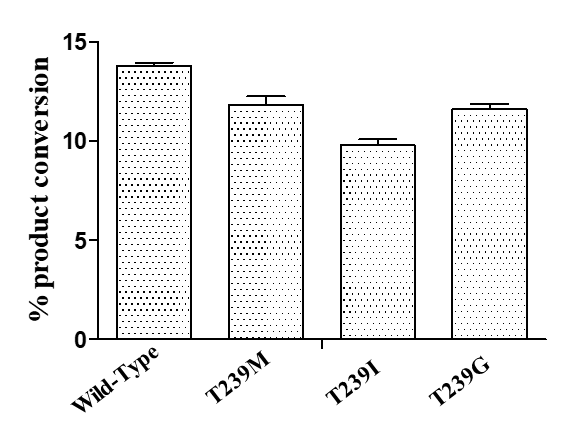


**Figure S2**. 2β- hydroxytestosterone formation by different mutants. The reaction was catalyzed in the presence of 40 mM H_2_O_2_, 200 µM testosterone, and 1 µM CYP.


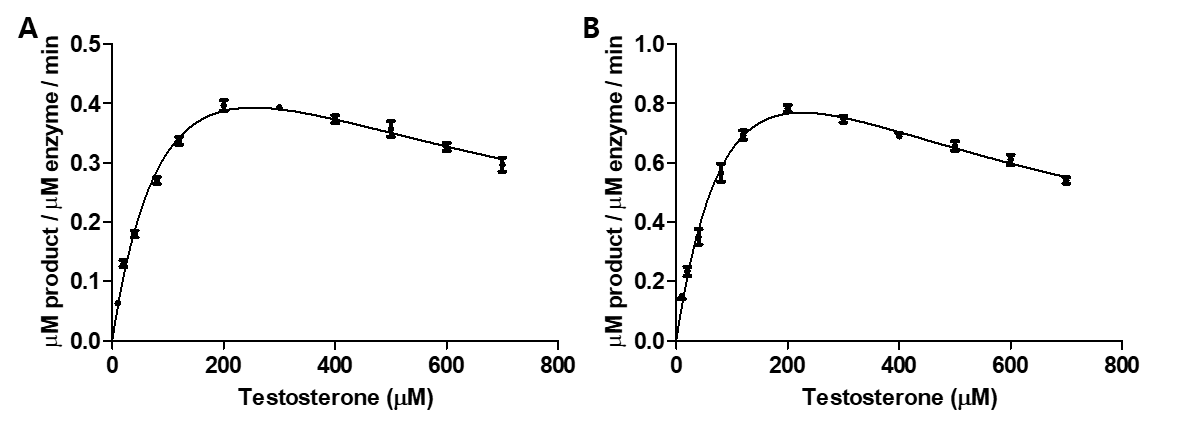


**Figure S3**. Hyperbolic fit for 2β- hydroxytestosterone formation. **A**. Substrate inhibition kinetics for 2β- hydroxytestosterone formation by CYP105D18 using GOx/glucose system. **B**. Substrate inhibition kinetics for 2β- hydroxytestosterone formation by F184A mutant of CYP105D18 using GOx/glucose system. All the data were generated from the three independent reactions using 1 µM enzyme, 36 mM glucose, and 8 U/L GOx.
